# The value of FT4/TSH ratio in the differential diagnosis of Graves’ disease and subacute thyroiditis

**DOI:** 10.3389/fendo.2023.1148174

**Published:** 2023-06-15

**Authors:** Yingjie Zhang, Yu Wang, Miao Liu, Lingge Wei, Jianmin Huang, Ziqian Dong, Meichao Guan, Weijie Wu, Jianqing Gao, Xiaojie Huang, Xin Guo, Peng Xie

**Affiliations:** ^1^ Department of Nuclear Medicine, The Third Hospital, Hebei Medical University, Shijiazhuang, Hebei, China; ^2^ Department of Nuclear Medicine, Zhangjiakou First Hospital, Zhangjiakou, Hebei, China

**Keywords:** thyrotoxicosis, FT4/TSH ratio, Graves’ disease, subacute thyroiditis, differential diagnosis

## Abstract

**Objective:**

To explore the value of the FT4/TSH ratio in the etiological diagnosis of newly diagnosed patients with thyrotoxicosis.

**Methods:**

The retrospective study was conducted on 287 patients with thyrotoxicosis (122 patients with subacute thyroiditis and 165 patients with Graves’ disease) and 415 healthy people on their first visit to our hospital. All patients underwent thyroid function tests including the measurement of T3, T4, FT3, FT4, TSH, T3/TSH, and T4/TSH. The receiver operating characteristic (ROC) curve was employed to evaluate the value of FT4/TSH in the differential diagnosis of Graves’ disease and subacute thyroiditis, and compared with other related indicators.

**Results:**

The area under the curve of FT4/TSH for diagnosing Graves’ disease and thyroiditis was 0.846, which was significantly larger than the area under the curve of T3/T4 ratio (*P*< 0.05) and FT3/FT4 ratio (*P*< 0.05). When the cut-off value of the FT4/TSH ratio was 5731.286 pmol/mIU, the sensitivity was 71.52%, the specificity was 90.16%, the positive predictive value was 90.77% and the negative predictive value was 70.06%. The diagnostic accuracy was 79.44%.

**Conclusion:**

FT4/TSH ratio can be used as a new reference index for the differential diagnosis of thyrotoxicosis.

## 1 Introduction

Thyrotoxicosis is a series of clinical syndromes characterized by a rapid increase in thyroid hormones in the blood caused by abnormal thyroid function in patients (1). Thyrotoxicosis can be divided into two types according to the different causes: hyperthyroidism and non-hyperthyroidism. The most common cause of thyrotoxicosis due to hyperthyroidism is Graves’ disease (GD), and the most common cause of thyrotoxicosis due to non-hyperthyroidism is subacute thyroiditis (ST) (1–4).

GD is an autoimmune disease. The etiology is primarily due to the presence of circulating anti-thyroid-stimulating-hormone receptor (TSH-R) stimulating autoantibodies in the body, which leads to hyperthyroidism (5). The clinical manifestations are not limited to the thyroid gland but are a syndrome involving multiple systems, including hypermetabolic symptom, diffuse goiter, Graves’ ophthalmopathy (GO), pretibial myxedema (PTM), and thyroid acromegaly. Hypermetabolic symptom including heat sensitivity, excessive sweating, weight loss, and fatigue. Menstrual cycle disturbances can occur in female and erectile dysfunction in male. ST, also known as granulomatous thyroiditis, giant cell thyroiditis, is the result of an inflammatory thyroid process of unknown etiology (6). The most common clinical manifestations include neck pain with tenderness and general fatigue. It is worth noting that compared with GD, ST can also show weight loss, heat sensitivity and hyperhidrosis.

In clinical work, there are great similarities between GD and ST in terms of clinical presentations and laboratory thyroid hormone levels. However, significantly different treatments should be chosen due to their different etiologies. Therefore, how to further differentiate GD and ST becomes a question worthy of our consideration, and differentiating GD and ST has an important guideline for the treatment of thyrotoxicosis. We usually make the diagnosis based on the patient’s clinical symptoms, and laboratory examinations, combined with the results of the radioactive iodine uptake (RAIU) test and conventional ^99m^TcO4 thyroid scintigraphy. It is important to note that there are limitations to each of these tests (1). Firstly, subacute thyroiditis usually has a history of upper respiratory tract infection, but in some patients with subacute thyroiditis, there is no history of upper respiratory tract infection or neck pain and other evidence of inflammatory infection. Secondly, thyrotropin receptor antibodies (TRAb) are positive on laboratory tests in GD patients, but in some GD patients, the test is negative. Thirdly, radiological iodine uptake experiments and thyroid imaging are of great clinical value. However, these two tests are radiological and are not permitted during pregnancy and lactation (7). Finally, many regions, especially community hospitals do not have the conditions to carry out nuclear medicine department examinations.

There are some difficulties in the differential diagnosis of patients with subacute thyroiditis and Graves’ disease. It is necessary to find a clinical differential diagnosis method that is easy to carry out, rapid, accurate, and applicable to all patients. Laboratory testing has been used in recent years as an easy, rapid, and accurate differential diagnostic method that applies to all patients. Some previous studies have been used in diagnosing GD and ST, including the ratio of total triiodothyronine(T3)/total thyroxine(T4) (8), the ratio of free triiodothyronine (FT3)/free thyroxine (FT4). There are few studies on the value of the FT4/thyroid-stimulating hormone (TSH) ratio. This study aimed to evaluate the usefulness of the ratio of FT4/TSH for differentiating GD from ST, we anticipate discovering a new indicator for the etiology of thyrotoxicosis.

## 2 Materials and methods

### 2.1 Patients

The Institutional Review Board of The Third Hospital, Hebei Medical University approved this retrospective study (Approval No.: W2022-003-1).

A total of 287 patients with thyrotoxicosis who visited the endocrinology clinic of our hospital for the first time from January 1, 2019, to January 31, 2020, were enrolled. In addition, 415 healthy subjects were selected as the healthy control group at the same time.

The inclusion criteria were as follows: (1) having signs and symptoms of thyrotoxicosis;(2) diffuse thyroid lesions on palpation or color doppler ultrasonography;(3) serum TSH level decreased, with or without changes in serum FT3 and FT4 levels; (4) patients first visit due to thyrotoxicosis without any treatment; (5) patients who can complete the thyroid radioactive iodine uptake (RAIU) test.

The exclusion criteria were as follows:(1) thyrotoxicosis caused by other causes was finally determined (such as pregnancy, multinodular toxic goiter, toxic adenoma, amiodarone-induced thyrotoxicosis and/or exogenous thyroxine intake); (2) concomitant diseases that may affect the measurement of thyroid function;(3) patients with hyperthyroidism complicated with thyroiditis;(4) patients with any one of FT3, FT4, TSH higher or lower than the measured value range.

### 2.2 Methods

#### 2.2.1 Thyroid function laboratory tests

Peripheral venous blood was collected from all subjects. All the subjects were collected in the morning on an empty stomach, and the specimens were collected from the procoagulant tube containing separation glue. After collection, the specimens were allowed to stand for more than 30 minutes and then centrifugation for 10 minutes at 3500r/min. Specimen examination was completed within 2 hours after sample collection, and the test was carried out by the standard operating procedures of our laboratory. At the same time, low-value and high-value quality controls are used to manage the quality of results. Automated chemiluminescent immunoassays (ADVIA centaur XP; Siemens) were used to determine Serum levels of TSH, FT3, FT4, T3, and T4, and the quality control products were used Bio-Rad. The corresponding reference ranges for serum FT3, FT4, TSH, T3 and T4 were 3.5-6.5pmol/L, 11.5-22.7pmol/L, 0.55-4.78mIU/L, 0.92-2.79 nmol/L and 58.1-161 nmol/L, respectively.

#### 2.2.2 The radioactive iodine uptake test

All subjects were required to undergo radioactive iodine uptake (RAIU) test to confirm the diagnosis, and the examination process was carried out according to the standard examination process of RAIU test. The final diagnosis report is jointly issued by 2 nuclear medicine physicians.

#### 2.2.3 Diagnostic criteria

According to the 2018 European Thyroid Association Guideline for the Management of Graves’ Hyperthyroidism (9) and related references (10). The diagnostic criteria of Graves’ disease were as follows: (1) the patient had symptoms and signs of hypermetabolism caused by thyrotoxicosis; (2) diffuse thyroid enlargement (confirmed by palpation and ultrasound), with some cases lacking goiter; (3) laboratory examination showed that the serum T4 level was increased, and the serum TSH level was decreased; (4) exophthalmos and other invasive eye signs; (5) the patient had pretibial myxedema; (6) laboratory tests showed that the TRAb was positive. (7) increased ^131^I uptake in radioactive iodine uptake (RAIU) test or increased radioactive uptake in ^99m^TcO4 thyroid scintigraphy. Among them, 1-3 items are essential for diagnosis, and 4-7 items can further provide the basis for the determination of the etiology.

According to the United States Guidelines for the diagnosis and treatment of thyroid diseases (1, 11) and related contents (12), the diagnostic criteria for the thyrotoxicosis stage of ST were as follows: (1) hypermetabolic symptoms and signs of thyrotoxicosis, with or without systemic symptoms of acute inflammation; (2) mild to moderate thyroid enlargement, moderate hardness, obvious or no obvious tenderness, (3) increased serum T4 level, decreased TSH level; (4) decreased ^131^I uptake in RAIU test or decreased radioactive uptake in ^99m^TcO_4_ thyroid scintigraphy.

Confirmation of diagnosis of Graves’ disease and subacute thyroiditis, two experienced physicians (one from the department of endocrinology and the other from the department of nuclear medicine) were invited to independently make a diagnosis of Graves’ disease and subacute thyroiditis based on all clinical data and the diagnostic criteria developed in this study. If the diagnosis was consistent, the diagnosis was established. In cases of disagreement, the diagnosis was made by a third experienced physician.

### 2.3 Statistical analysis

SPSS 25.0 statistical software was used for data statistics. Normal distribution samples were expressed as mean ± standard deviation (X ± SD), and a one-way analysis of variance was used for the comparison of multiple groups of samples. The results of skewed distribution samples were expressed as median M (interquartile range Q), and the Kruskal-Walis H test was used for the comparison of multiple samples. MedCal 19 statistical software was employed to analyze the ROC curve of the data, and the Area Under Curve (AUC), cut-off value, sensitivity, specificity, positive predictive value, negative predictive value, and diagnostic accuracy of Graves’ disease and subacute thyroiditis were determined. *P*<0.05 was considered statistically significant.

## 3 Results

### 3.1 The basic information of the included population was collected

A total of 702 samples were included in this study, including 415 healthy people, 186 males, and 229 females. There were 122 patients with subacute thyroiditis, including 15 males and 107 females. There were 165 patients with Graves’ disease, including 39 males and 126 females. The average age was 45.50 ± 12.15 in the healthy population, 50.23 ± 15.93 in the subacute thyroiditis group, and 48.18 ± 14.86 in the Graves’ disease group. There was no significant difference in age among the three groups (*P*=0.206).

**Figure 1:**
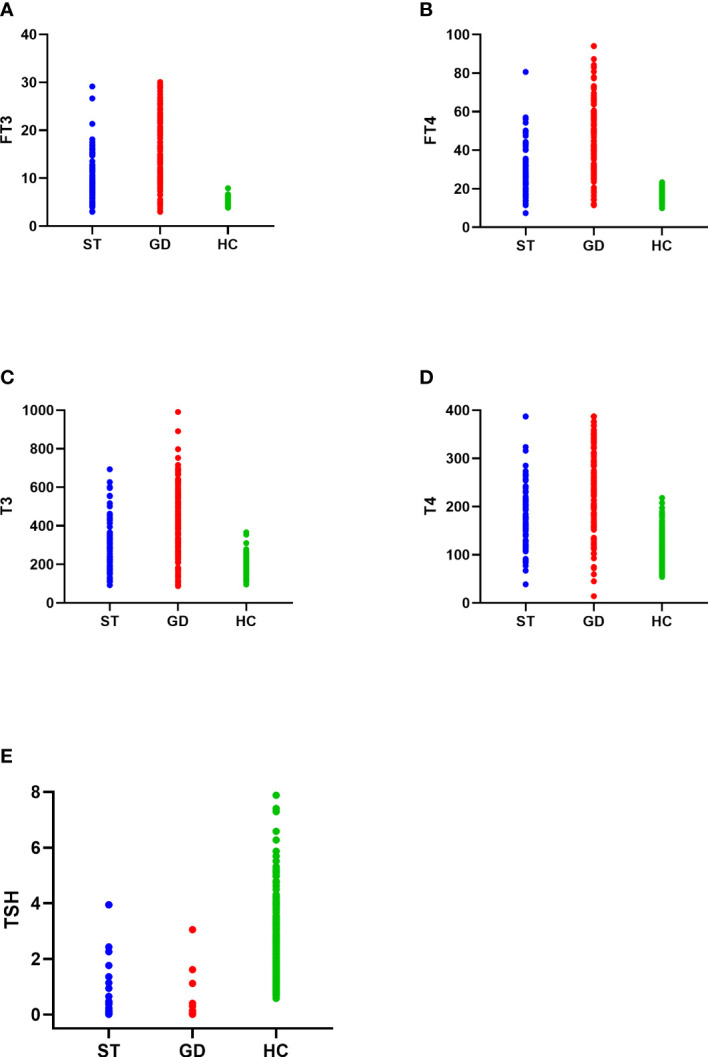
The distribution of FT3 **(A)**, FT4 **(B)**, T3 **(C)**, T4 **(D)**, and TSH **(E)** in the normal control group (HC), subacute thyroiditis group (ST) and Graves’ disease group (GD), respectively.

The FT3, FT4, TSH, T3, and T4 levels of the normal control group, ST group, and GD group were as follows: (1)FT3(5.174(0.693) pmol/L, 7.824(4.316) pmol/L, and 15.708(11.327) pmol/L); (2)FT4(16.254(3.096) pmol/L, 25.478(12.707) pmol/L, and 43.086(27.864) pmol/L); (3)TSH(1.886(1.356) mIU/L, 0.021(0.065) mIU/L, and 0.005(0.005) mIU/L); (4)T3(174.636(42.135) nmol/L, 235.374(163.509) nmol/L, and 435.527(257.981) nmol/L); (5)T4(107.070(28.380) nmol/L, 163.830(88.688) nmol/L, and 241.230(136.934) nmol/L). The differences in the levels of FT3, FT4, TSH, T3, and T4 in the three groups were statistically significant (H=374.473, 362.789, 474.602, 277.105, 304.929; *P*<0.001). The levels of FT3, FT4, TSH, T3, and T4 were significantly different between the normal control group and the ST group (*P*<0.001), the normal control group and the GD group (*P*<0.001), and the ST group and the GD group (*P*<0.001) (**Table 1** and **Figure 1**).

**Table 1:** Baseline characteristics in the patients with Graves’ disease, subacute thyroiditis, and healthy control.

Total	HC	ST	GD
	415	122	165
Sex(Male/Female))	186/229	15/107	39/126
Age (years)	45.5 ± 12.15	50.23 ± 15.93	48.18 ± 14.86
FT_3_(pmol/L)	5.174(0.693)	7.824(4.316)*	15.708(11.327)*^#^
FT_4_(pmol/L)	16.254(3.096)	25.478(12.707) *	43.086(27.864) *^#^
TSH(mIU/L)	1.886(1.356)	0.021(0.065) *	0.005(0.005) *^#^
T_3_(nmol/L)	174.636(42.135)	235.374(163.509) *	435.527(257.981) *^#^
T_4_(nmol/L)	107.070(28.380)	163.830(88.688) *	241.230(136.934) *^#^
FT_3_/FT_4_	0.321(0.063)	0.325(0.071)	0.361(0.086) *^#^
T_3_/T_4_	1.643(0.434)	1.529(0.671) *	1.773(0.548) *^#^
T_3_/TSH	89.317(78.361)	10830.913(28360.613)*	82621.000(131762.583)*^#^
T_4_/TSH	53.978 (48.978)	7413.676(19114.200)*	46440.000(60474.893)*^#^
FT_4_/TSH (pmol/mIU)	8.755(6.030)	1,307.200(3,622.580) *	8,292.857(12,177.600) *^#^

*Compared with healthy control group, *P*<0.05, #Compared with subacute thyroiditis group, *P*<0.05.

Normal control group (HC); Subacute thyroiditis group (ST); Graves’ disease group (GD).

**Figure 2:**
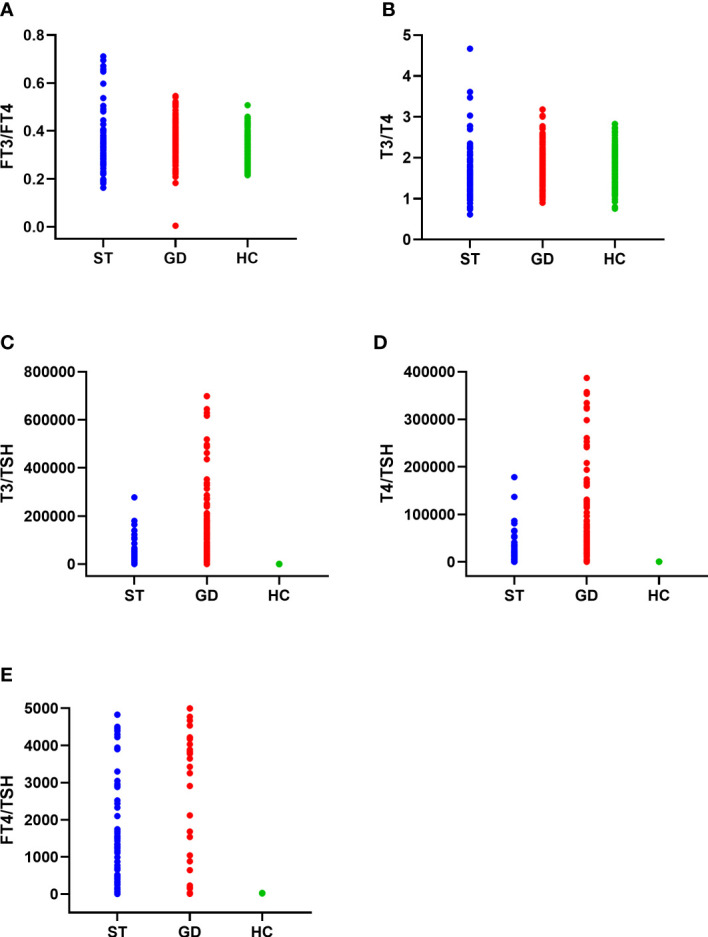
The distribution of FT3/FT4 ratio **(A)**, T3/T4 ratio **(B)**, T3/TSH ratio **(C)**, T4/TSH ratio **(D)**, and FT4/TSH ratio **(E) **in the normal control group (HC), subacute thyroiditis group (ST) and Graves’ disease group (GD), respectively.

The FT3/FT4, T3/T4, and FT4/TSH ratio of the normal control group, ST group, and GD group were as follows: (1) FT3/FT4(0.321(0.063) pmol/mIU, 0.325(0.071) pmol/mIU, and 0.361(0.086) pmol/mIU); (2) T3/T4(1.643(0.434) pmol/mIU, 1.529(0.671) pmol/mIU, and 1.773(0.548) pmol/mIU); (3) FT4/TSH (8.755(6.030) pmol/mIU, 1,307.200(3,622.580) pmol/mIU, and 8,292.857(12,177.600) pmol/mIU). The differences in FT3/FT4, T3/T4, and FT4/TSH ratios in the three groups were statistically significant (H=45.122, 27.496, 472.014; *P*<0.001). There was no significant difference in FT3/FT4 ratio between the normal control group and the ST group (*P*=1.000). However, there were significant differences in FT3/FT4 ratio between the normal control group and the GD group (*P*<0.001) and between the ST group and the GD group (*P*<0.001). There were significant differences in T3/T4 ratio and FT4/TSH ratio between the above 3 groups (*P*<0.001) (**Table 1** and **Figure 2**).

The T3/TSH, and T4/TSH ratio of the normal control group, ST group, and GD group were as follows: (1) T3/TSH(89.317(78.361) pmol/mIU, 10830.913(28360.613) pmol/mIU, and 82621.000(131762.583)pmol/mIU); (2) T4/TSH(53.978(48.978)pmol/mIU, 7413.676(19114.200)pmol/mIU, and 46440.000(60474.893) pmol/mIU). The differences in T3/TSH and T4/TSH ratios in the three groups were statistically significant (H=469.785, 472.620; *P*<0.001). The levels of T3/TSH ratio and T4/TSH ratio were significantly different between the normal control group and the ST group (*P*<0.001), the normal control group and the GD group (*P*<0.001), and the ST group and the GD group (*P*<0.001) (**Table 1** and **Figure 2**).

**Figure 3:**
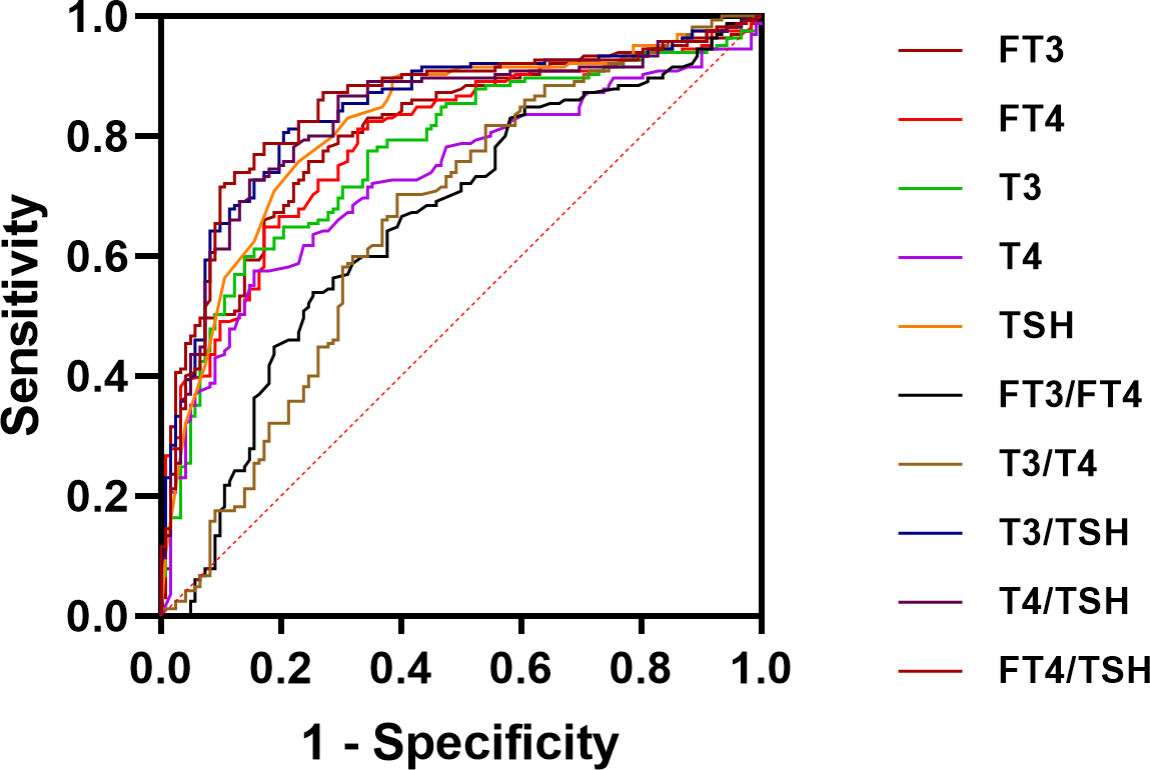
ROC curve analysis for FT3, FT4, T3, T4, TSH, FT3/FT4 ratio, T3/T4 ratio, T3/TSH ratio, T4/TSH ratio, and FT4/TSH ratio.

Our study results by plotting ROC curves, the ROC area of FT3, FT4, TSH, T3, T4, FT3/FT4 ratio, T3/T4 ratio, T3/TSH ratio, T4/TSH ratio and FT4/TSH ratio in the differential diagnosis of Graves’ disease and subacute thyroiditis was 0.806, 0.790, 0.818, 0.771, 0.727, 0.654, 0.658,0.843,0.831 and 0.846, respectively (**Figure 3**). Notably, the AUC of the FT4/TSH ratio was larger than that of all other parameters, and the differences were statistically significant (*P*<0.05). The cut-off value of 5731.286 pmol/mIU for FT4/TSH ratio for differential diagnosis of subacute thyroiditis and Graves’ disease had a diagnostic sensitivity of 71.52%, specificity of 90.16%, a positive predictive value of 90.77%, a negative predictive value of 70.06%, and diagnostic accuracy of 79.44% (**Table 2**).

**Table 2:** Results of each parameter in the differential diagnosis of Graves’ disease and subacute thyroiditis.

Variables	AUC	95%CI	cut-off value	Sensitivity (%)	Specificity (%)	Positive Predictive Value(%)	Negative Predictive Value(%)	Diagnostic Accuracy(%)
FT3	0.806*	0.756 - 0.850	>10.641	75.76	75.41	79.62	69.23	74.91
FT4	0.790*	0.738 - 0.836	>29.025	81.21	67.21	77.01	72.57	75.26
TSH	0.818*	0.768 - 0.861	≤0.008	75.76	77.05	81.70	70.15	76.31
T3	0.771*	0.718 - 0.818	>369.877	60.00	86.07	84.62	61.18	70.73
T4	0.727*	0.672 - 0.778	>220.59	57.58	84.43	83.33	59.54	68.99
FT_3_/FT_4_	0.654*	0.596 - 0.709	>0.353	53.94	74.59	72.95	53.94	62.02
T_3_/T_4_	0.658*	0.599 - 0.712	>1.593	70.30	60.66	70.73	60.16	66.20
T_3_/TSH	0.843	0.795-0.883	>36419.289	80.61	79.51	84.18	75.20	80.14
T_4_/TSH	0.831	0.782-0.872	>27520	72.73	85.25	86.96	69.80	78.05
FT_4_/TSH	0.846	0.799 - 0.886	>5731.286	71.52	90.16	90.77	70.06	79.44

* Comparison with FT4/TSH ratio, the difference in area distribution under the ROC curve is considered statistically signiﬁcant. *P*<0.05.

## 4 Discussion

As the study of Graves’ disease and subacute thyroiditis progress, clinicians need to get some new clinical diagnostic indicators with the aim of better differential diagnosis. We are always looking for quick and easy methods to help diagnose thyroid disease correctly. With the advances in thyroid hormone testing methods in recent years, we have been able to study thyroid hormone levels more comprehensively, especially in terms of thyroid hormone ratios. As early as 1978, a related study concluded that the ratio of T3/T4 can be used to distinguish Graves’ disease from thyroiditis. T3/T4 ratio has been considered an ancillary tool in delineating the etiology of thyrotoxicosis, with T3/T4 >20ng/mg suggesting hyperthyroidism (1, 8). However, T3 and T4 are the products of the combination of hormones and thyroid-binding globulin (TBG). Their levels are directly affected by plasma proteins, so theoretically the accuracy of TT3 and TT4 is limited. Currently, it is more commonly used to replace TT3 and TT4 with FT3 and FT4, which are less influential factors (13–15). There are various related studies showed that the value of the FT3/FT4 ratio in determining the etiology of thyrotoxicosis (13, 16). Wu (17) et al. ‘s study suggested that FT3/FT4 ratio was a better indicator for the differential diagnosis of Graves’ disease and subacute thyroiditis, and the area under the ROC curve was 0.86 (95%CI: 0.84-0.88), the cut-off value was 1.99pmol/mIU, the sensitivity was 79%, and the specificity was 80%. Recent studies have shown that inferior thyroid artery blood flow, T3/T4 ratio and FT3/FT4 ratio are useful parameters in the differentiation between Graves’ disease and Destructive thyroiditis (DT) (18). The three parameters in combination yielded a positive predictive value of 100% in the diagnosis of Graves’ disease. The results of the FT3/FT4 ratio in the differential diagnosis of Graves’ disease in this study are not ideal. The reason for this result may be the elevated levels of FT3 and FT4 in the patient’s serum due to thyrotoxicosis caused by ST. The degree of elevation is similar to the level of FT3 and FT4 elevation caused by GD. Therefore, in our study, the results of the FT3/FT4 ratio differential diagnosis of Graves’ disease need further justification. Yoshimura Noh et al. (19) concluded that there is some overlap between the FT3/FT4 ratios of patients with painless thyroiditis and those of patients with GD. When FT4 levels are much higher than FT3, this ratio can be helpful in distinguishing between painless thyroiditis and GD. Among the thyroid-based studies, the aspect of the FT3/FT4 ratio and TT3/TT4 ratio is a hot topic for scientific researchers. However, the findings of the FT3/FT4 ratio and TT3/TT4 ratio in the differential diagnosis of GD and thyroiditis are not yet uniform. Therefore it is important to find new indicators for a more reliable diagnosis (19–22).

This study demonstrated the value of FT4/TSH in the diagnosis of Graves’ disease and subacute thyroiditis. The area under the ROC curve for the differential diagnosis of Graves’ disease and subacute thyroiditis was 0.846. When the cut-off value for the differential diagnosis of Graves’ disease and subacute thyroiditis was 5731.286 pmol/mIU, the sensitivity of the diagnosis was 71.52%, the specificity was 90.16%, the positive predictive value was 90.77%, the negative predictive value was 70.06%, and the diagnostic accuracy was 79.44%. In our study, ROC analysis of the T3/TSH ratio and T4/TSH ratio showed that their AUC were 0.843(95% confidence interval: 0.795-0.883) and 0.831(95% confidence interval: 0.782-0.872), which were lower than the AUCs of the TRAb level and FT4/TSH ratio (**Table 2**). The FT4/TSH ratio can be used as a reference indicator for the differential diagnosis of Graves’ disease and has great advantages in reflecting thyroid function. On the one hand, the characteristics of FT4 are such that it is not influenced by changes in plasma protein concentration. On the other hand, FT4 is the first to show feedback regulation fluctuations in the presence of abnormal thyroid function. However, FT4 may fluctuate to a lesser extent and overlap with the normal range, so it is necessary to use TSH as a ratio to improve the diagnostic positivity. TSH is sensitive to changes in feedback from thyroid hormones. With the advances in detection techniques, we can find a significant difference between the degree of TSH decreases in Graves’ disease and that in thyroiditis. Studies have shown a 2-fold change in FT4 and an approximately 100-fold change in TSH values (23). The increase of FT4 and its negative feedback inhibition of TSH can be used to comprehensively judge the changes in thyroid hormones. Overall, we use the FT4/TSH ratio as a new indicator to differentiate Graves’ disease from subacute thyroiditis.

There were several limitations in this study, First, the sample size is small, and we need to enlarge the study considering a greater number of patients in order to provide further validation of the results. Second, GD and ST can be directly diagnosed in most cases, we should demonstrate the usefulness of the FT4/TSH ratio in the diagnosis of patients with GD without TSH receptor antibodies and patients with ST with insignificant thyroid tenderness and/or weak systemic inflammatory symptoms in the future.

It should be noted that 20 samples were deleted according to the exclusion criteria. The samples were deleted because FT4 was greater than the upper limit (≥12 ng/dl, 2 samples) or TSH was below the lower limit of detection measurement (≤0.001 ulU/ml, 18 samples) and the ratio could not be calculated. According to our follow-up survey, these 20 samples were found to be finally diagnosed as patients with Graves’ disease. Corollary to this, we suggest that Graves’ disease is more likely to be diagnosed when FT4 values are extremely high or TSH values are extremely low. The FT4/TSH ratio is more instructive only when FT4 is not yet reached the upper limit of detection and the TSH is not yet reached the lower limit of detection.

The FT4/TSH ratio can be used as a new indicator for the differential diagnosis of Graves’ disease and subacute thyroiditis. The FT4/TSH ratio can provide a basis for the correct clinical diagnosis of the disease, especially if the patient is unable to perform radioactive tests such as the radioactive iodine uptake (RAIU) test or ^99m^TcO_4_ thyroid scintigraphy, or the hospital is unable to perform these tests.

## Data availability statement

The original contributions presented in the study are included in the article/supplementary material. Further inquiries can be directed to the corresponding author.

## Author contributions

YW, PX, and LW contributed to the conception and design of the study. JH and WW organized the database. MG, ZD, and XH performed the statistical analysis. ML and YZ wrote the first draft of the manuscript. XG, JG, YW, and PX wrote sections of the manuscript. All authors contributed to the article and approved the submitted version.
